# Exogenous prostaglandin D_2_ as a modulator in bovine endometritis: implications for reducing antibiotic use in dairy cattle

**DOI:** 10.3389/fvets.2025.1618203

**Published:** 2025-08-12

**Authors:** Xiaolin Yang, Shuangyi Zhang, Bo Liu, Lili Guo, Pengfei Gong, Jingze Wu, Yi Zhao, Wei Mao, Jinshan Cao

**Affiliations:** ^1^Key Laboratory of Clinical Diagnosis and Treatment Techniques for Animal Disease, Ministry of Agriculture, Inner Mongolia Agricultural University, Hohhot, China; ^2^Laboratory of Veterinary Clinical Pharmacology, College of Veterinary Medicine, Inner Mongolia Agricultural University, Hohhot, China; ^3^Inner Mongolia Bayannaoer City Municipal Center for Disease Control and Prevention, Linhe, China

**Keywords:** endometritis, *Escherichia coli*, prostaglandin D2, bone marrow-derived, endometrial tissue

## Abstract

**Introduction:**

Bovine endometritis is a common postpartum uterine infection that significantly impacts the health and production performance of dairy cows, leading to economic losses for farms. Bovine endometritis is closely associated with pathogenic microorganisms, disturbances in uterine microecology, and localized inflammatory damage. *Escherichia coli* (*E. coli*) is the primary pathogenic bacterium responsible for bovine endometritis. Prostaglandin D_2_ (PGD_2_) is abundant in the uterine environment. However, its role in *E. coli*-induced endometritis remains largely unknown. We used bovine bone marrow-derived macrophages (BMDMs) and bovine endometrial tissue to investigate the specific genes and molecular mechanisms involved in *E. coli*-induced bovine endometritis.

**Methods and results:**

Transcriptomic data show that *E. coli* infection significantly upregulated 2,141 genes and downregulated 2,381 genes in bovine BMDMs. *E. coli* activates various molecular functions in bovine BMDMs, with the most closely related being the inflammatory response, in which Prostaglandin-Endoperoxide Synthase 2 (PTGS2) plays a crucial role. Additionally, ELISA analysis revealed that *E. coli* infection significantly promoted the secretion of PGD_2_ in BMDMs. In the early stage of infection, ELISA results showed that exogenous PGD_2_ significantly promoted the secretion of TNF-α, IL-1β, and IL-6 in BMDMs and endometrial tissues, suggesting its role in enhancing the inflammatory response during early infection. Further q-PCR and immunofluorescence analyses demonstrated that PGD_2_ markedly upregulated the expression of damage-associated molecules, including high mobility group box 1 (HMGB-1) and hyaluronic acid-binding protein 2 (HABP-2). In addition, immunofluorescence and MTT assay results indicated that PGD_2_ enhanced the intracellular survival of *E. coli* in macrophages. H&E staining showed that PGD_2_ exacerbated pathological damage in bovine endometrial tissues. Contrastingly, at later stages, PGD_2_ suppresses the expression of inflammatory mediators, decreases *E. coli* survival, and alleviates tissue damage.

**Discussion:**

These results not only deepen our understanding of the multifaceted role of exogenous PGD2 in uterine pathophysiology but also provide potential therapeutic implications for the treatment of bovine endometritis.

## 1 Introduction

Endometritis is a common bacterial infection affecting the reproductive system of cattle globally. It severely impacts bovine the reproductive efficiency, hinders animal welfare, and can delay or prevent successful pregnancies (1). *Escherichia coli* (*E. coli*) is the primary pathogen associated with this condition, with bacterial colonization occurring in the uterus of 80–100% of cows within 2 weeks postpartum (2). The reported incidence of *E. coli*-induced endometritis during this period is 49.2% (3). Postpartum, the uterus is highly vulnerable to infection by *E. coli*, leading to increased inflammation triggered by microbe- and damage-associated molecular patterns (DAMPs) (4). *E. coli* infection also contributes to cell death, tissue damage, and necrosis (5), posing a significant threat to the health and recovery of postpartum cows, while reducing the economic efficiency of dairy farming (6). Consequently, strategies to protect the uterus from *E. coli* infection are of growing research interest.

At present, the primary clinical approaches for managing bovine endometritis involve the use of antibiotics and non-steroidal anti-inflammatory drugs (NSAIDs) (7, 8). However, long-term or inappropriate antibiotic use can lead to the development of antibiotic-resistant bacterial strains. This resistance not only makes the treatment of subsequent infections more difficult but may also significantly reduce the effectiveness of antibiotics, thereby posing a serious challenge to herd health management (9–11). In addition, the issue of antibiotic residues in meat and dairy products cannot be overlooked. Antibiotic residues may trigger allergic reactions in humans and contribute to the development of antibiotic resistance, thereby weakening the body's immune defense mechanisms and posing a potential threat to human health (12–14). NSAIDs are another commonly used treatment for bovine endometritis, primarily acting by reducing inflammation, alleviating pain, and improving clinical symptoms (15–17). However, NSAIDs inhibit the synthesis of all prostaglandins, which can interfere with the normal tissue repair processes in the uterus and delay the healing of the endometrial lining (18, 19). Consequently, it is crucial to identify novel therapeutic targets that can provide effective treatment while reducing the risk of adverse effects.

The imbalance between infection and self-defense mechanisms results in postnatal reproductive diseases, including puerperal inflammation, clinical endometritis, subclinical endometritis, etc. (20). Endometritis affects over 45% of cows within 3 weeks postpartum, with 15–20% developing clinical endometritis and 30% developing subclinical endometritis (6). Upon *E. coli* invasion, it adheres to and colonizes the mucosal surface of the endometrium, particularly in areas with tissue damage (21). Endometrial epithelial and mesenchymal cells detect DAMPs via innate immune receptors, triggering the release of cytokines, chemokines, and prostaglandins that amplify the inflammatory response (22). These mediators recruit and activate neutrophils and macrophages, which aid in eliminating pathogens and resolving tissue damage (23). Macrophages play a key role in immune responses, facilitating phagocytosis, clearing infections, and regulating inflammation to promote tissue repair and restore homeostasis (24). Monocytes and macrophages in the bone marrow and peripheral blood primarily originate from hematopoietic stem cells in the bone marrow (25). As primary immune cells, they retain the key physiological functions of tissue-resident macrophages. Moreover, under specific induction conditions, bone marrow-derived macrophages (BMDMs) can be reliably polarized into either M1 or M2 phenotypes (26), making them an ideal *in vitro* model for studying macrophage-mediated inflammatory responses during *E. coli* infection in dairy cows. Based on these advantages, BMDMs were selected as the experimental model in this study. Cytokines, chemokines and prostaglandins (PGs) are recognized as key regulators of *E. coli*-induced endometritis (27). However, the mechanism by which PGs influence this process in bovine endometritis remains unclear.

PGs, members of the eicosanoid family of lipid compounds, play critical roles in regulating inflammation and immune responses. The major PGs include Prostaglandin D_2_ (PGD_2_), E_2_ (PGE_2_), H_2_ (PGH_2_), and I_2_ (PGI_2_). PGD_2_ is synthesized via the catalytic activities of cyclooxygenases and PGD_2_ synthases in mast cells, macrophages, and other cellular sources (28). PGD_2_ has been implicated in microbial infections, immunomodulation, inflammation during cancer progression, and renal injury (29). PGD_2_ mediates its biological effects through two G protein-coupled receptors, DP_1_ and DP_2_ (28). However, the pathophysiological role of PGD_2_ remains controversial. Some studies have highlighted its pro-inflammatory properties, showing that it enhances immune cell chemotaxis and accumulation at inflammation sites, exacerbating conditions such as inflammatory bowel disease, rhinitis, and asthma (30–32). Conversely, other research highlights its anti-inflammatory functions, demonstrating that PGD_2_ signaling suppresses inflammasome hyperactivation, providing protection against *Helicobacter pylori*-induced gastritis, acute lung inflammation, and brain inflammation (33–35). Intriguingly, PGD_2_ exhibits anti-inflammatory effects in colitis, but paradoxically promotes carcinogenesis during its resolution phase (30). Recent studies have also provided insight into PGD_2′_s dual role in inflammatory conditions. For instance, our research group demonstrated that exogenous PGD_2_ enhances *E. coli* induced inflammatory responses in mouse macrophages (36). Additionally, the PGD_2_-DP_1_ pathway may exert protective effects against endometritis in dairy cows (37). Hence, the paradoxical role of PGD_2_ has attracted attention; however, its role in bovine endometritis remains largely unexplored.

Therefore, this study aims to explore novel therapeutic targets, with the goal of providing new strategies for the treatment of bacterial bovine endometritis. To this end, this study employed transcriptomics to select inflammation-related genes associated with *E. coli* infection in BMDMs. Using bovine BMDMs and endometrial tissue as models, we conducted a comprehensive and systematic investigation of the molecular mechanisms through which PGD_2_ modulates *E. coli*-induced bovine endometritis.

## 2 Materials and methods

### 2.1 Ethical statement

All animal experiments adhered to the regulations stipulated in the Administration of Affairs Concerning Experimental Animals in China and received approval from the Animal Welfare and Research Ethics Committee of Inner Mongolia Agricultural University (Approval ID: NND2021013).

### 2.2 Bacterial strains

The *E. coli* O157:H7 strain used in this study was purchased from Bena Culture Collection (BNCC186579, Beijing, China). A 1 ml suspension of *E. coli* O157:H7 strain (at a concentration of 1 × 10^7^ CFU) preserved in the laboratory was inoculated into 100 ml of Luria-Bertani (LB) broth (Oxoid, Basingstoke, LTD, UK). Incubate the culture at 37°C with shaking at 200 rpm for 12 h, or until the OD_600_ of the culture reaches 0.9. The bacterial suspension was serially diluted and plated onto LB agar. After incubation at 37°C for 18 h, colonies were counted, and the concentration was quantified as CFU/ml.

### 2.3 Infection of BMDMs and treatment *in vitro*

In this study, bovine rib samples were obtained from healthy adult Holstein cows at the Beiya Slaughterhouse in Hohhot, Inner Mongolia, China. All animals had passed veterinary health inspections and quarantine assessments, and were confirmed to be free of major diseases. Slaughter was conducted solely for commercial food production purposes. After slaughter at the abattoir, cow rib bones were collected immediately post-mortem, placed on ice, and transported to the laboratory for further processing. All bone marrow samples were processed within 1 hour of animal death to preserve cell viability and ensure reproducibility of the results. Bone marrow was extracted from cow ribs by flushing with phosphate-buffered saline (PBS; Hyclone, Logan, UT, USA). The collected cells were centrifuged at 2,900 g for 8 min, and the supernatant was discarded. The cells were subsequently resuspended in erythrocyte lysis buffer and incubated for 5 min to facilitate lysis. The cells were then centrifuged at 1,300 g for 8 min and subsequently cultured in RPMI 1,640 medium supplemented with 20% fetal bovine serum (Hyclone, Logan, UT, USA) and 20 ng/ml M-CSF (Kingfisher Biotech Inc., USA) at 37°C in a 5% CO_2_ atmosphere. After 7 days, unattached cells were removed, and adherent cells were used for the experiments. To induce M1 macrophages, cells (2 × 10^6^ per well) were treated with 1 μg/ml of lipopolysaccharide for 24 h, followed by an 8-h resting period. M1 BMDMs were confirmed by immunofluorescence (Supplementary Figure 1).

### 2.4 Experimental animals and treatment

The tissues used in this study were obtained from animals slaughtered at the Beiya Slaughterhouse in Hohhot, Inner Mongolia, China. Prior to slaughter and tissue collection, all animals underwent health screening, including tests for common reproductive diseases. To minimize the potential impact of underlying infections on the study results, only animals that met these health standards were selected. The experiment selected 30 healthy Holstein dairy cows aged 15–18 months, and fresh uterine horn samples were collected (~400 kg). All samples were collected from sexually mature cows in the proestrus phase of the estrous cycle. Endometrial tissue culture was established following the method of Li (5). After rinsing the uterine horns 3 times with PBS containing 100 IU/ml penicillin, 100 IU/ml streptomycin, and 2.5 mg/ml amphotericin B, the uterine horns were incubated at 4°C for 1 h. Under aseptic conditions, the uterine horns of cows were longitudinally incised, and small pieces of endometrial tissue measuring 2 × 2 mm were excised. These explants were then randomly allocated to six-well plates for culture.

### 2.5 Experimental infection and treatment *in vitro*

BMDMs were randomly divided into four groups: Control, PGD_2_-treated, *E. coli*-infected, and PGD_2_ + *E. coli* co-treatment groups. In the PGD_2_-treated and co-treatment groups, cells were pretreated with PGD_2_ at a final concentration of 1 × 10^−6^ M (1 × 10^−6^ M; Cayman Chemical Company, Ann Arbor, MI, USA) for 24 h prior to infection (see Supplementary Figure 2 for concentration selection). Subsequently, *E. coli* was added at a multiplicity of infection (MOI) of 5:1 for further experimentation. After 1 h of infection with 100 μg/ml tobramycin to remove extracellular bacteria, the medium was changed to continue the culture (38).

The bovine endometrial tissues were randomly assigned to the following experimental groups: Control, PGD_2_ (1 × 10^−5^ M), PGD_2_ (4 × 10^−9^ M), *E. coli*, PGD_2_ (1 × 10^−5^ M) + *E. coli*, and PGD_2_ (4 × 10^−9^ M) + *E. coli*. Prior to infection, tissues in the PGD_2_ treatment groups were pretreated with the corresponding concentration of PGD_2_ for 24 h. Subsequently, infection was performed using *E. coli* at a concentration of 1 × 10^6^ CFU/ml. Each group was set up in three independent wells, with tissue samples obtained from different individual cows to enhance biological reproducibility. In addition, each well was analyzed in triplicate, and the average value was used for subsequent data analysis.

### 2.6 RNA isolation, library preparation and transcriptome analysis

BMDMs were treated with PGD_2_ (1 × 10^−6^ M) for 24 h, followed by infection with *E. coli* at a MOI of 5:1 for 4 h. Total RNA was isolated from the cells using TRIzol reagent. The RNA concentration and integrity were then evaluated using a NanoDrop spectrophotometer (Thermo Scientific, USA) to confirm that the RNA remained intact. The libraries were constructed following the manufacturer's instructions, after which transcriptome sequencing was performed and the data analyzed by OE Biotech Co (Shanghai, China). Gene counts for each sample were normalized using DESeq2 software, with expression levels estimated based on the baseMean value. The fold changes were calculated, and significance was assessed using a negative binomial distribution test (NB test). The differentially expressed genes (DEGs) selected met the criteria of |log2 Fold Change| > 1.5 and a significance *P*-value < 0.05. Significantly enriched genes and related pathways were identified through GO and KEGG enrichment analysis and their main biological functions were explored (with a significance threshold of *P* < 0.05). Finally, further enrichment analysis of GO and KEGG pathways was conducted using the OECloud tool (https://cloud.oebiotech.com/task/).

### 2.7 q-PCR

Cells were collected at 2, 4, and 6 h post-*E. coli* infection. Total RNA was extracted using the Axygen RNA Kit (Axygen Scientific, USA), and RNA was reverse-transcribed into cDNA using the PrimeScript RT kit (Vazyme, Nanjing, China). The mRNA levels of various genes including Prostaglandin-Endoperoxide Synthase 2 (*PTGS2*), *HMGB-1*, and *HABP-2* were quantified using the SYBR Green Master Mix Kit (Roche Applied Science, Mannheim, Germany). Gene expression was normalized according to actin mRNA levels, and data were analyzed using the 2^−^^Δ*ΔCt*^ method (39). All primers information is in Table 1.

**Table 1:** Primer sequences used for real-time PCR.

**Gene**	**Primer sequence (5^′^to 3^′^)**	**Annealing temperature, °C**	**Product size, bp**
*β-Actin*	F: ATCGGCAATGAGCGGTTC R: CCGTGTTGGCGTAGAGGT	60	144
*PTGS2*	F: CTCCTGTGCCTGATGACTGC R: TGGTCCTCGTTCAAAATCTGTCT	60	196
*HMGB-1*	F: AAGTTCAAGGATCCCAATGCAC R: GCTTATCATCCGCAGCAGTGT	60	162
*HABP-2*	F: TCTGACAACCCTGACTGGTACTAC R: GTGGTAAGGAGGACTCTGAGTAATG	60	212

### 2.8 Molecular docking analysis

We performed molecular docking analysis of the drug PGD_2_ with the key target protein PTGDR. After removing all associated ligands, the three-dimensional structures of PGD_2_ and PTGDR were obtained from PubChem and the Protein Data Bank (PDB), respectively. AutoDock v1.5.7 was then used for ligand preparation, including the removal of water molecules, processing of nonpolar hydrogen atoms, and identification of the active binding site. Docking conformations were calculated using AutoDock Vina, and the optimal docking model was selected. Finally, the key binding amino acids and optimal binding conformation were visualized using the PyMOL molecular graphics system v2.0 in the Python environment (40).

### 2.9 Enzyme-linked immunosorbent assay

The concentrations of PGD_2_, TNF-α, IL-1β, IL-6, IL-8 and IL-10 in the supernatants of BMDMs and endometrial tissue were measured using bovine PGD_2_ (Cayman Chemical Company, Ann Arbor, MI, USA), TNF-α and IL-6 (R&D Systems, Minneapolis, MN, USA), and IL-1β, IL-8, and IL-10 (Kingfisher Biotech, St. Paul, MN, USA) ELISA kits according to the manufacturer's instructions.

### 2.10 Western blot analysis

Total protein was extracted from treated cells using the M-PER mammalian protein extraction reagent (Thermo Scientific, Waltham, MA, USA), and protein concentrations were determined using the BCA Protein Assay Kit (Thermo Scientific, Rockford, IL, USA). A total of 10 μg of protein from each sample was separated using a 12% SDS-PAGE gel, and the proteins were subsequently transferred to a polyvinylidene fluoride (PVDF) membrane for Western blot analysis. Subsequently, the membrane was blocked with StartingBlock™ (TBS) blocking buffer (Thermo Fisher, MA, USA) at room temperature for 1 h, followed by incubation with primary antibodies at 4°C for 14 h. The primary antibodies employed were antiphospho-ERK, anti-ERK, antiphospho-p38, anti-p38, antiphospho-NF-κB p65, anti-NF-κB p65 (Cell Signaling Technology, 1:1,000 dilution), and anti-GAPDH (1:10,000) monoclonal antibodies. Immunoreactive bands were visualized via chemiluminescence using horseradish peroxidase-conjugated secondary anti-rabbit and anti-mouse antibodies along with chemiluminescent substrate (Thermo Scientific). The band density on the blots was quantified using ImageJ software (National Institutes of Health, Bethesda, MD, USA).

### 2.11 Cell viability assay

Cell viability and intracellular *E. coli* survival were assessed via the MTT assay. BMDMs were seeded in 96-well plates at a density of 1 × 10^4^ cells per well, with 180 μl of culture medium added to each well, and cultured at 37°C in 5% CO_2_. After 24 h of PGD_2_ treatment, cells were infected with *E. coli* at an MOI of 5:1 for 2.5 and 6 h. Cell viability was evaluated using the MTT assay following the guidelines provided by the manufacturer (Solarbio, Beijing, China).

### 2.12 Phagocytosis and bacterial killing of *E. coli* by BMDMs

To investigate the impact of PGD_2_ on phagocytosis and bacterial killing, BMDMs were cultured at a density of 2 × 10^6^ cells per 35 mm glass-bottom Petri dish. After treatment, cells were labeled with 8 μm 11′-dioctadecyl-3,33′3′-tetramethylindocarbocyanine perchlorate (DiI, Thermo Scientific). After labeling *E. coli* with Hoechst 33258 dye for 30 mins, BMDMs were infected for 30 mins, 2.5 h, and 6 h at 37°C. The cells were then fixed with 4% paraformaldehyde and imaged using a confocal microscope (LSM 800; Carl Zeiss, Oberkochen, Germany) at × 400 magnification.

### 2.13 Immunofluorescence and histological analysis

Immunofluorescence analysis of endometrial tissues from dairy cows was performed using established methods (41). After frozen sections, they were incubated overnight at 4°C using primary antibodies (1:100 dilution) to HMGB-1 and HABP-2. After blocking, tissue sections were incubated with donkey anti-rabbit IgG-Alexa Fluor 647 fluorescent secondary antibody (1:1000 dilution; Abcam) for 1 h in the dark. Imaging and quantification of fluorescence intensity were conducted using confocal microscopy (LSM 800, Zeiss, Oberkochen, Germany) at × 400 magnification.

Sections were made by dehydration through an alcohol gradient (70%, 80%, 90%, 100%) and paraffin embedding.These sections were then stained with hematoxylin and eosin (H&E) and imaged using an Axio Scan Z1 slide scanner (Zeiss, Thornwood, NY, USA). All images were captured under identical conditions.

### 2.14 Statistical analysis

Data were analyzed using GraphPad Prism 8 (GraphPad Software, CA, USA), and results are presented as mean ± standard deviation (SD). Statistical significance was evaluated using one-way analysis of variance (ANOVA) with Tukey's multiple comparisons test or two-way ANOVA with Bonferroni's post-hoc test, depending on the experimental design. Differences were considered statistically significant when the *P* value was < 0.05 (^*^P < 0.05, ^**^P < 0.01, ^***^P < 0.001, and ^****^P < 0.0001).

## 3 Results

### 3.1 Altered gene levels in *E. coli*-infected BMDMs

**Figure 1:**
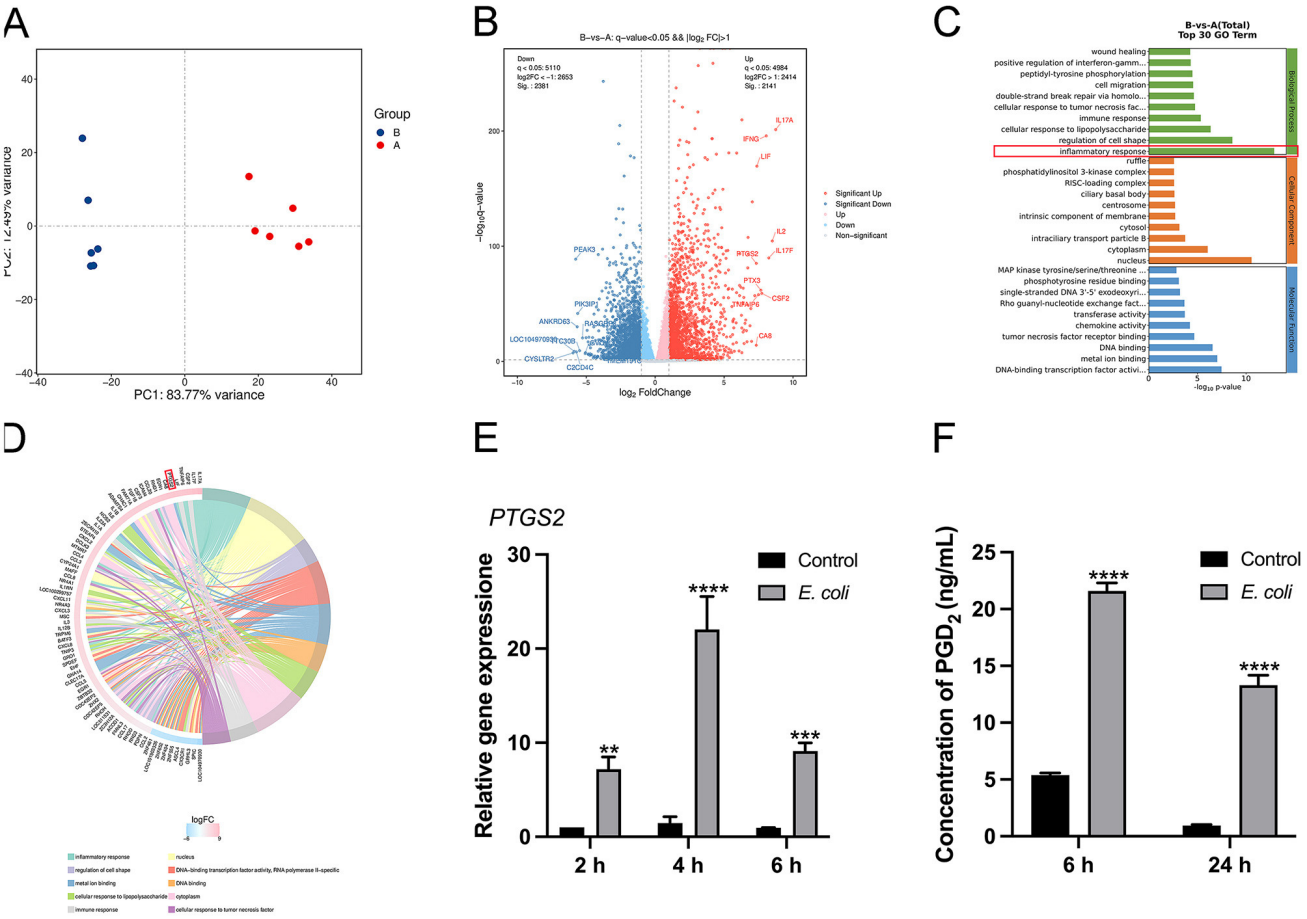
*Escherichia coli* induces *PTGS2* and PGD_2_ expression in BMDMs of dairy cows. **(A)** PCA score plots. A: control, B: *E. coli*. **(B–D)** The GO enrichment analysis and KEGG enrichment analysis results of genes in *E. coli*-induced BMDMs. **(E)** The mRNA expression of *PTGS2*. **(F)** Secretion of PGD_2_. Results were expressed as the mean ± SD of multiple independent experiments and analyzed by two-way ANOVA with Bonferroni's post-test (*n* = 3). *P < 0.05, **P < 0.01, ***P < 0.001, ****P < 0.0001.

Transcriptome sequencing was performed to identify potential target genes associated with *E. coli* infection in BMDMs. The principal component analysis (PCA) identified distinct differences among various sample groups based on transcriptome data, with PCA1 and PCA2 accounting for 83.77 % and 12.49 % of the total variation, respectively (Figure 1A). Transcriptomic analysis of *E. coli*-infected BMDMs identified 4,522 significantly dysregulated genes, including 2,141 upregulated and 2,381 downregulated genes, based on a *q*-value < 0.05 and |log2 fold change (FC)| > 1 (Figures 1A, B). Gene Ontology (GO) analysis revealed that the most significant differential gene expression was observed in the inflammatory response (Figure 1C). The key genes involved were *IL17A, IL17F, TNFAIP6*, and *PTGS2* (Figure 1D). Among these, we focused on *PTGS2*, which showed a significant increase in mRNA expression at 2, 4, and 6 h post-infection (Figure 1E, *P* < 0.01). In *E. coli*-infected macrophages, the secretion levels of PGD_2_ were elevated at 6 and 24 h (Figure 1F, *P* < 0.0001). These results indicate that PGD_2_ plays a critical role in the response of BMDMs to *E. coli* infection.

### 3.2 The effect of the drug PGD_2_ on BMDMs

**Figure 2:**
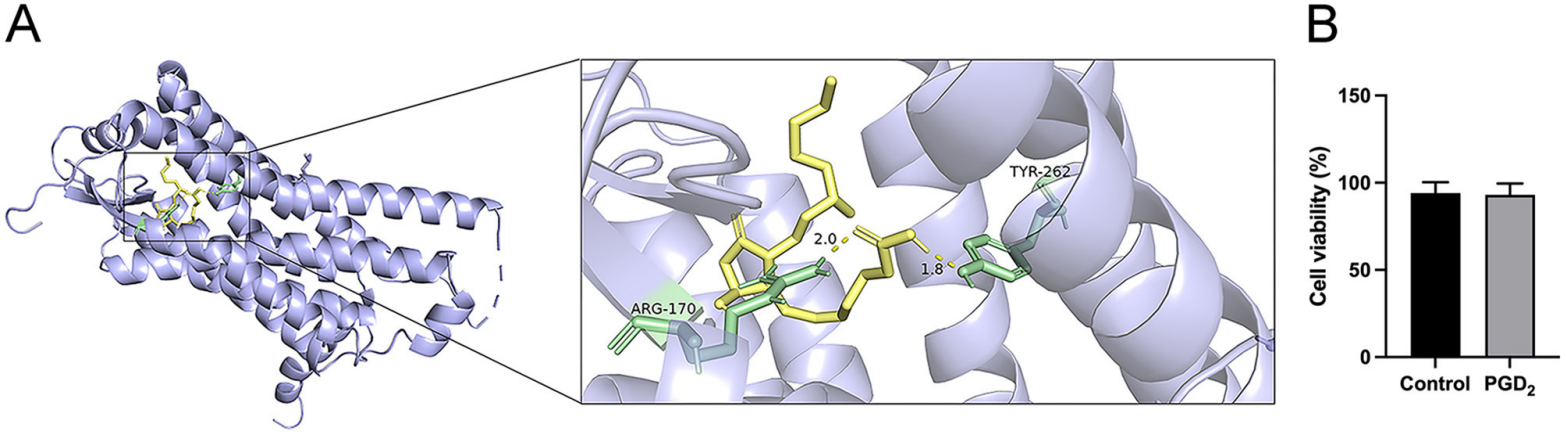
Effect of the drug PGD_2_ on the activity of BMDMs. **(A)** The molecular docking results. Amino acids are in pale green, with hydrogen bond distances shown as yellow dashed lines. **(B)** The effect of PGD_2_ on BMDM viability.

Molecular docking analysis of the interaction between PGD_2_ and the PTGDR-associated protein (PDB ID: 7M8W) revealed that PGD_2_ forms a stable binding with the PTGDR-associated protein, with a binding energy of −7.9 kcal/mol (Figure 2A). In addition, MTT assay results showed no significant difference in cell viability between PGD_2_-treated BMDMs for 24 h and the control group, indicating that PGD_2_ did not significantly impact macrophage viability (Figure 2B). These results indicate that PGD_2_ binds effectively to PTGDR and does not exert a significant effect on macrophage viability.

### 3.3 Effects of exogenous PGD_2_ on cytokine and chemokine production and inflammatory pathways during *E. coli* infection in BMDMs

**Figure 3:**
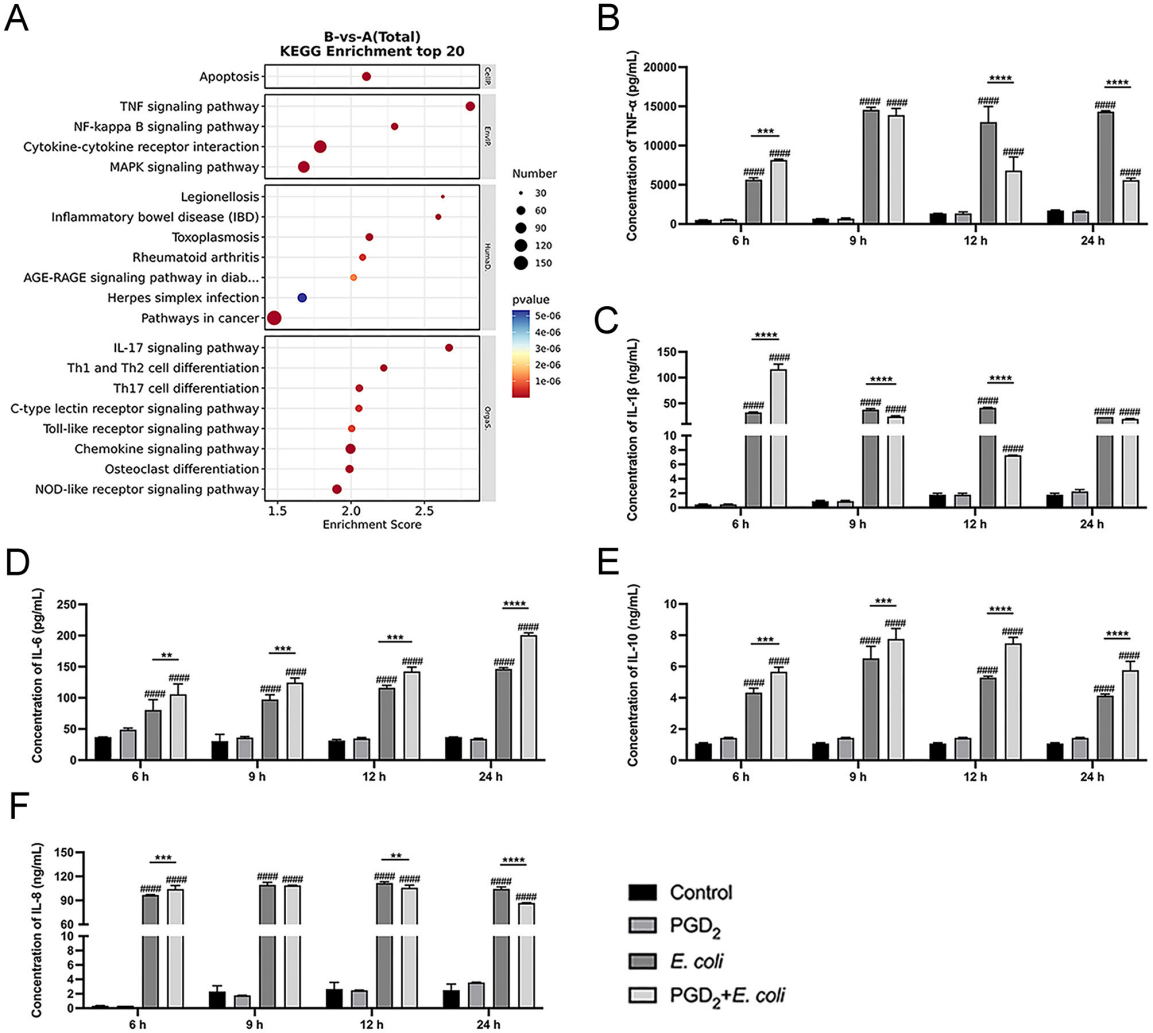
Effect of PGD_2_ on *Escherichia coli* induced cytokine secretion in BMDMs. **(A)** Top 20 KEGG pathway enrichment of upregulated genes in *E. coli*-infected BMDMs. **(B–F)** Secretion of TNF-α, IL-1β, IL-6, IL-10 and, IL-8. Results are expressed as mean ± SD of three independent experiments and were analyzed using two-way ANOVA with Bonferroni's post-hoc test. ^#^*P* < 0.05, ^##^*P* < 0.01, ^###^*P* < 0.001 and ^####^*P* < 0.0001 compared to control group. *P < 0.05, **P < 0.01, ***P < 0.001 and ****P < 0.0001 indicate statistically significant differences between two experimental groups.

Based on KEGG enrichment analysis, differentially expressed genes were significantly enriched in multiple inflammation-related signaling pathways, such as TNF signaling pathway, NF-κB pathway, cytokine-cytokine receptor interaction, and MAPK signaling pathway (Figure 3A). Therefore, we measured the secretion of cytokines and chemokine using ELISA and analyzed the activation of NF-κB and MAPK signaling pathways by Western blot. The ELISA results showed that PGD_2_ pretreatment significantly increased the secretion of TNF-α, IL-1β, IL-6, IL-8, and IL-10 in BMDMs at 6 h post-*E. coli* infection. However, at 12 and 24 h post-infection, PGD_2_ significantly downregulated the secretion of TNF-α, IL-1β, and IL-8 compared to the *E. coli* infection group, while the secretion of IL-6 and IL-10 remained elevated (Figures 3B–F, *P* < 0.01).

**Figure 4:**
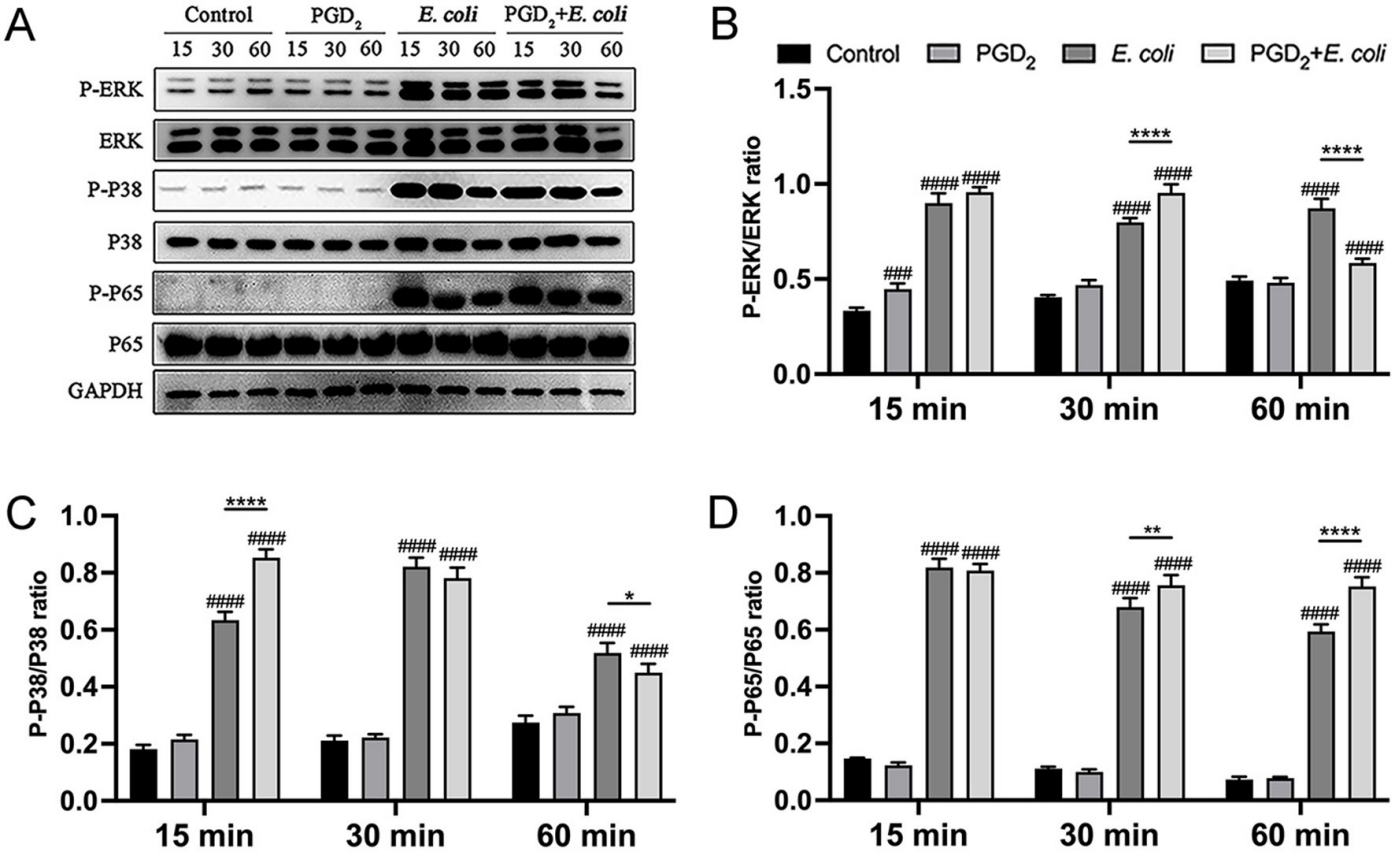
Effect of PGD_2_ on the activation of BMDMs signaling pathway induced in *Escherichia coli*. Phosphorylation of ERK, p38, and p65 was assessed by western blotting at 15, 30, and 60 min post-infection, with GAPDH as the loading control. Grayscale values were quantified using ImageJ software. Results are expressed as mean ± SD of three independent experiments and were analyzed using two-way ANOVA with Bonferroni's post-hoc test. ^#^*P* < 0.05, ^##^*P* < 0.01, ^###^*P* < 0.001 and ^####^*P* < 0.0001 compared to control group. *P < 0.05, **P < 0.01, ***P < 0.001 and ****P < 0.0001 indicate statistically significant differences between two experimental groups.

In addition, PGD_2_ treatment significantly enhanced the phosphorylation levels of MAPK (ERK, P38) and NF-κB (P65) in *E. coli*-infected BMDMs at 15 and 30 min (Figure 4, *P* < 0.01). However, at 60 min post-infection, PGD_2_ attenuated the phosphorylation levels of MAPK in BMDMs (Figure 4, *P* < 0.05). These findings suggest that PGD_2_ may influence the activation of macrophage MAPK and NF-κB signaling pathways during *E. coli* infection, thereby affecting the secretion of pro-inflammatory cytokines and chemokines.

### 3.4 Impact of exogenous PGD_2_ on BMDMs phagocytosis and intracellular killing

**Figure 5:**
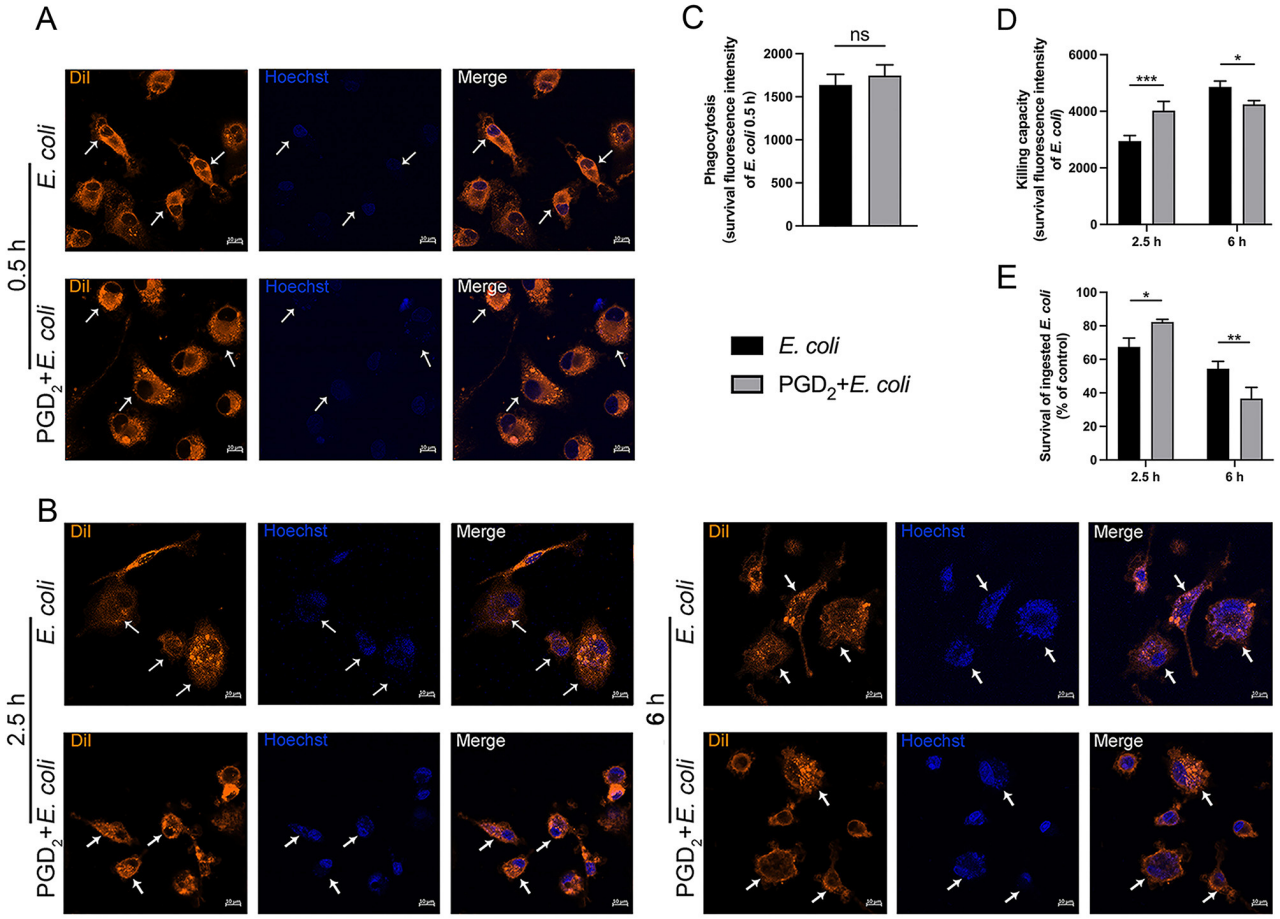
Effect of exogenous PGD_2_ on BMDMs phagocytosis and intracellular killing of *Escherichia coli*. **(A, B)** Phagocytosis and intracellular killing of Hoechst 33258-labeled *E. coli* (blue) within DiI-labeled BMDMs (orange) were analyzed via microscopy (×400, scale label = 10 μm). **(C)** The effect of PGD_2_ on *E. coli* phagocytosis by BMDMs. **(D)** The impact of PGD_2_ on *E. coli* killing by BMDMs. **(E)** The bacterial killing capacity of macrophages was quantified by a tetrazolium dye reduction assay. Results were expressed as the mean ± SD of multiple independent experiments and analyzed by two-way ANOVA with Bonferroni's post-test. *P < 0.05, **P < 0.01, ***P < 0.001. ns, not significant.

The effect of PGD_2_ on the ability of BMDMs to phagocytose and kill *E. coli* was assessed using Dil-labeled BMDMs and Hoechst-stained *E. coli*. After 0.5 h post-infection, no significant differences were observed, suggesting that PGD_2_ does not affect the phagocytic ability of BMDMs (Figures 5A, C). After 2.5 h of infection, the fluorescence intensity of bacteria in the PGD_2_ was higher in the treatment group than the *E. coli* infection group, indicating that PGD_2_ reduced the killing ability of BMDMs. However, after 6 h of infection, the fluorescence intensity of bacteria in the PGD_2_ treatment group was lower, suggesting that PGD_2_ enhanced the killing ability of BMDMs against *E. coli* (Figures 5B, D, *P* < 0.05). The effect of PGD_2_ on *E. coli* survival within macrophages was evaluated through the MTT assay. Consistent with these observations, PGD_2_ demonstrated a significant increase in the survival of internalized *E. coli* in macrophages (Figure 5E, *P* < 0.05). Together, these results suggest that PGD_2_ decreased intracellular killing in macrophages.

### 3.5 Effects of exogenous PGD_2_ on the expression of DAMPs in BMDMs and endometrial tissues of dairy cows infected with *E. coli*

**Figure 6:**
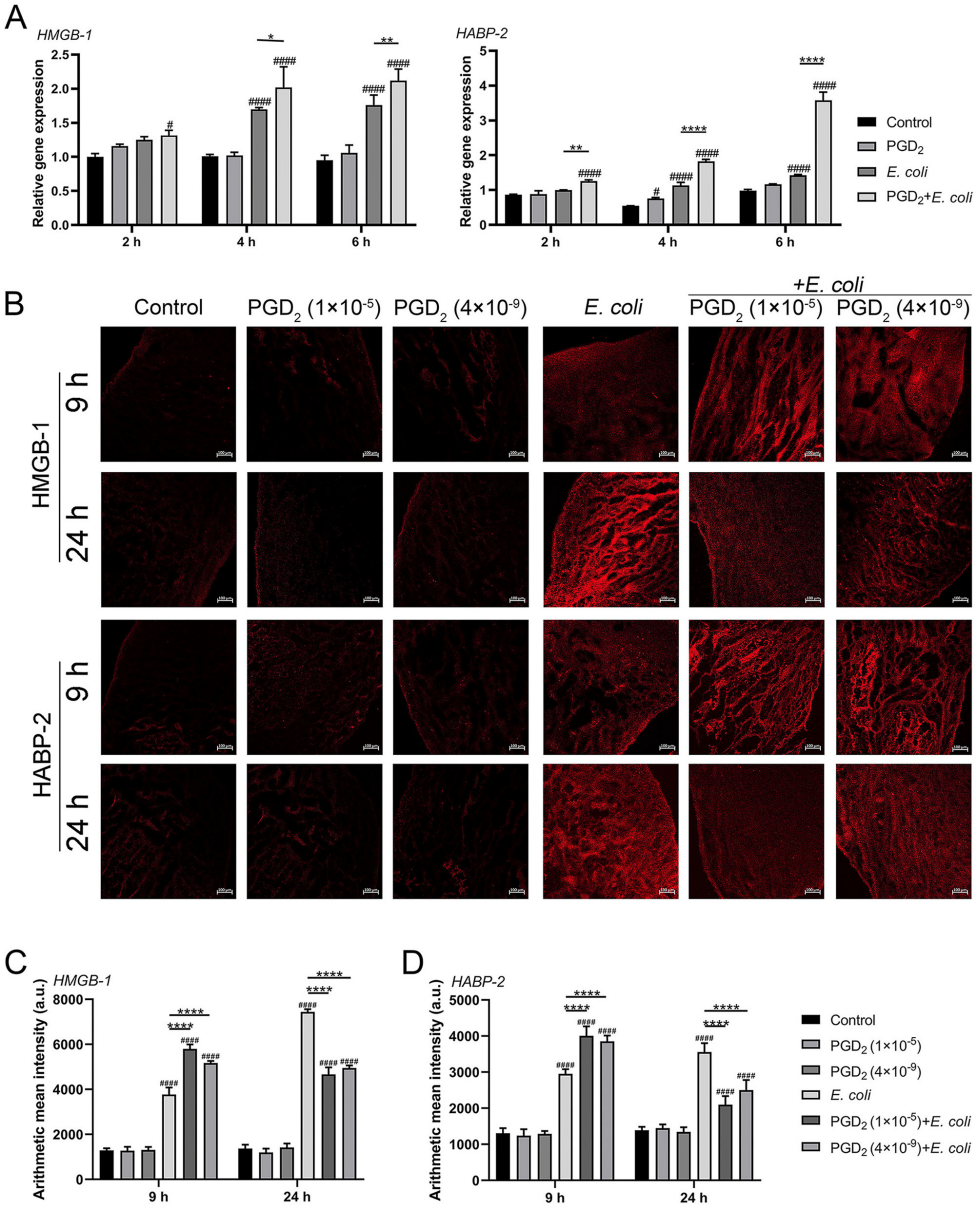
Effect of exogenous PGD_2_ on *Escherichia coli*-induced expression of *HMGB-1* and *HABP-2* in bovine BMDMs and endometrial tissues. **(A)** *HMGB-1* and *HABP-2* mRNA expression in BMDMs. **(B–D)** *HMGB*-*1* and *HABP*-*2* expression in *E. coli*-infected dairy cow endometrial tissue was assessed using ZEN software by immunofluorescence (Zeiss, × 100 magnification, scale label = 100 μm). Results are expressed as mean ± SD of three independent experiments and were analyzed using two-way ANOVA with Bonferroni's post-hoc test. ^#^*P* < 0.05, ^##^*P* < 0.01, ^###^*P* < 0.001 and ^####^*P* < 0.0001 compared to control group. *P < 0.05, **P < 0.01, ***P < 0.001 and ****P < 0.0001 indicate statistically significant differences between two experimental groups.

To determine the effect of PGD_2_ on the damage caused by *E. coli* infection in BMDMs and bovine endometrial tissues, we measured the expression of DAMPs (HMGB-1, HABP-2) using qPCR and immunofluorescence. Compared to the *E. coli* infection group, PGD_2_ significantly increased *HMGB-1* and *HABP-2* mRNA expression levels in BMDMs (Figure 6A, *P* < 0.05). Immunofluorescence staining of *E. coli*-infected endometrial explants showed that PGD_2_ treatment led to an increased expression of HMGB-1 and HABP-2 at 9 h post-infection (Figures 6B–D, *P* < 0.0001). However, this expression was decreased at 24 h (Figures 6B–6D, *P* < 0.0001). In conclusion, our data demonstrate that PGD_2_ modulates the release of HMGB-1 and HABP-2 in *E. coli*-infected endometritis in dairy cows, exhibiting distinct roles at different times.

### 3.6 Effect of exogenous PGD_2_ on histomorphometry of endometrium in *E. coli* infected cows

**Figure 7:**
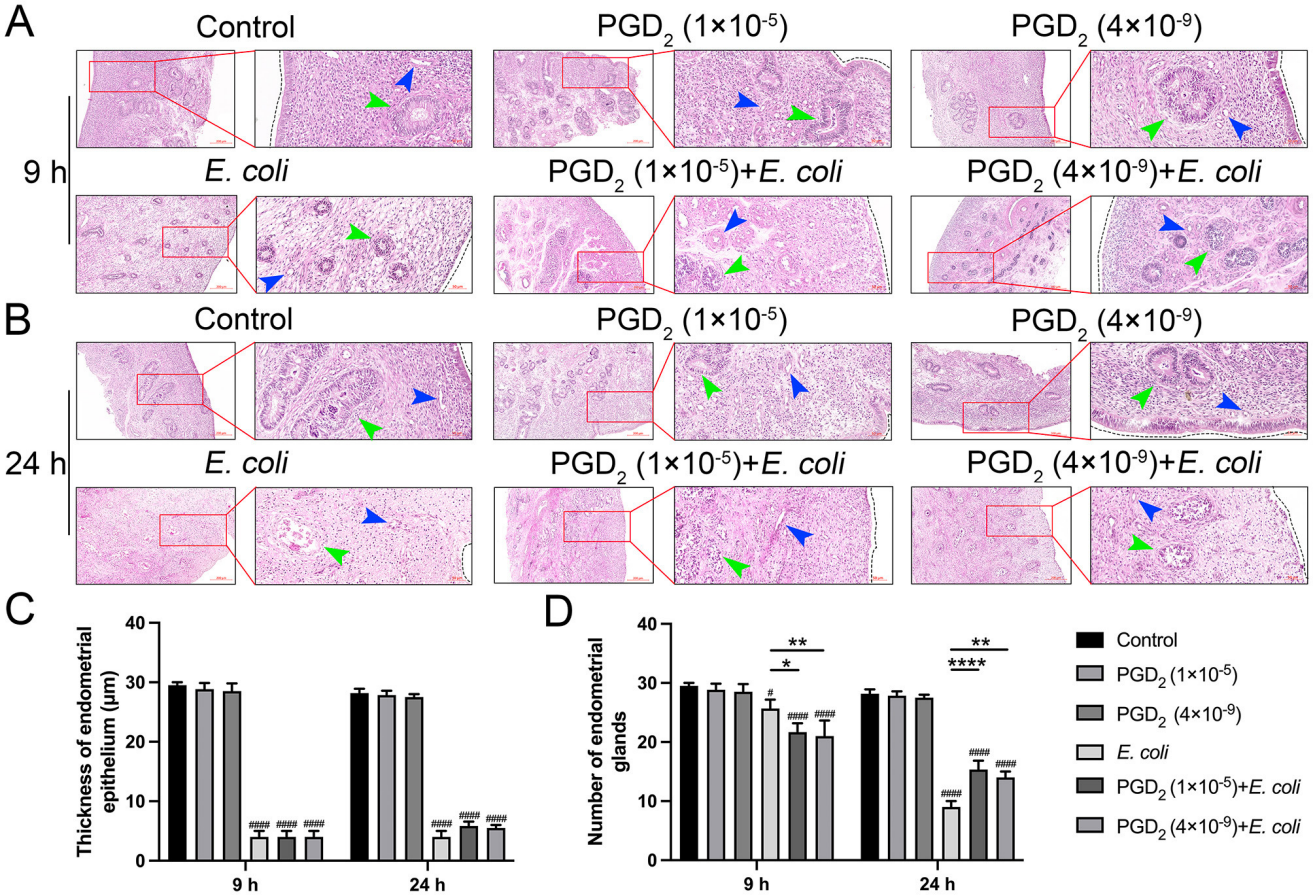
PGD_2_ attenuates *Escherichia coli*-induced endometrial tissue damage in dairy cows. **(A, B)** Micrographs of H&E-stained endometrial tissue sections. **(C)** Thickness of endometrial epithelium. **(D)** Number of endometrial glands. The black dotted line represents the endometrial epithelium, green arrows represent glands, and blue arrows represent the vessels. Scale label = 200 μm and 50 μm. Results are expressed as mean ± SD of three independent experiments and were analyzed using two-way ANOVA with Bonferroni's post-hoc test. ^#^*P* < 0.05, ^##^*P* < 0.01, ^###^*P* < 0.001 and ^####^*P* < 0.0001 compared to control group. *P < 0.05, **P < 0.01, ***P < 0.001 and ****P < 0.0001 indicate statistically significant differences between two experimental groups.

To gain more insight into the effects of PGD_2_ on uterine injury, we assessed histologic changes in the endometrium of dairy cows. Hematoxylin and Eosin (H&E) staining results demonstrate that both the control and PGD_2_-treated groups maintained intact endometrial structures, characterized by tightly arranged epithelial cells and well-defined glands and blood vessels. At 9 h post-infection, *E. coli*-infected tissues exhibited complete shedding of endometrial epithelial cells although glandular epithelial cells remained largely unaffected. PGD_2_-treated groups (1 × 10^−5^ M and 4 × 10^−9^ M) also showed endometrial epithelial cells shedding and loosening of glandular epithelial cells, some of which exhibited partial disintegration (Figure 7A). At 24 h post-infection, PGD_2_ treatment reduced endometrial damage, with the *E. coli*-infected group showing severe epithelial and glandular cell loss and necrosis, whereas the PGD_2_-treated groups retained relatively intact glandular and vascular structures (Figure 7B). Additionally, measurements of endometrial epithelial thickness and gland count (42) revealed no significant differences in epithelial thickness between the PGD_2_-treated and *E. coli*-infected groups at both 9 and 24 h. Notably, at 9 h post-infection, the PGD_2_-treated group showed a reduction in gland count compared to the *E. coli*-infected group, a trend that reversed by 24 h (Figures 7C, D, *P* < 0.05). These findings provide further evidence that PGD_2_ may play a dual role in *E. coli*-induced endometritis, aggravating tissue damage during the early phase of infection while contributing to tissue protection at later stages.

### 3.7 Regulation of inflammatory mediators by exogenous PGD_2_ in *E. coli*-infected endometrial tissues of cows

**Figure 8:**
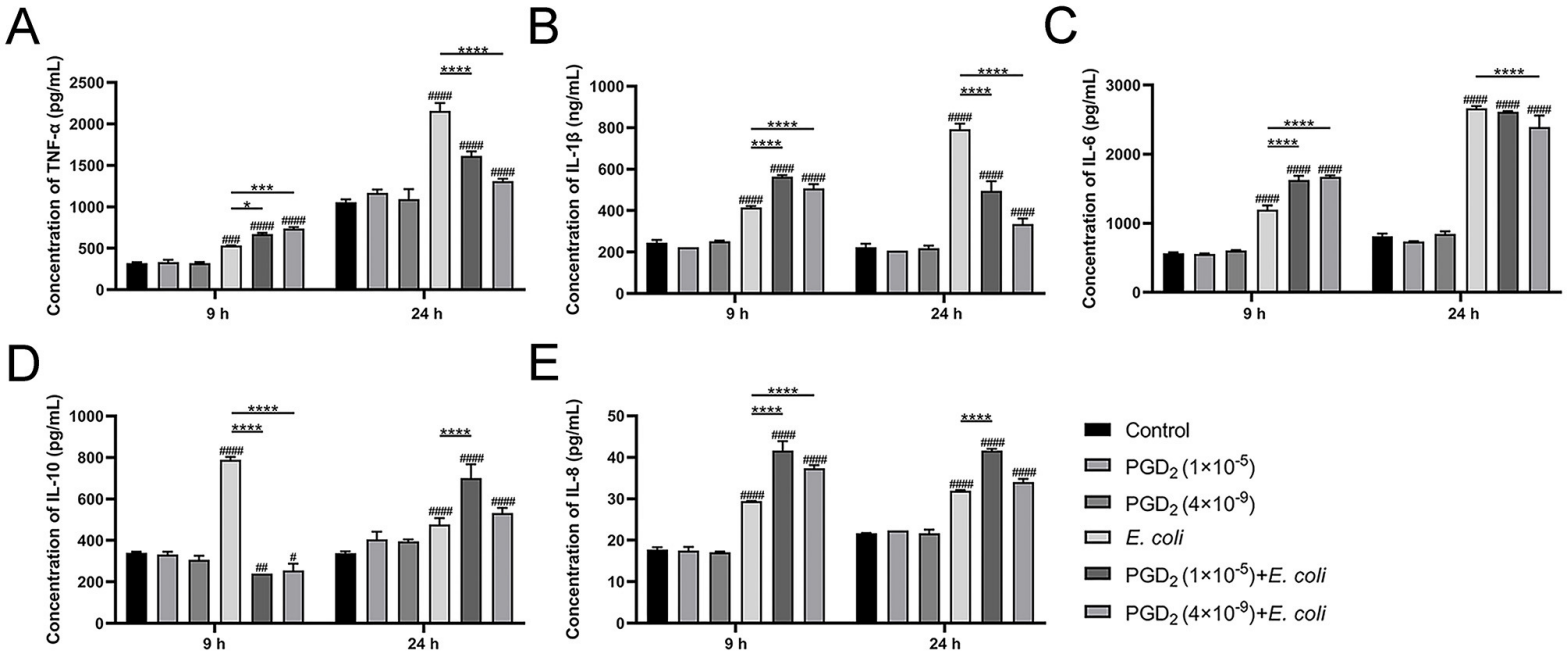
PGD_2_ modulates the level of *Escherichia coli*-induced inflammation in endometrial tissue of dairy cows. **(A)** TNF-α. **(B)** IL-1β. **(C)** IL-6. **(D)** IL-10. **(E)** IL-8. Results are expressed as mean ± SD of three independent experiments and were analyzed using two-way ANOVA with Bonferroni's post-hoc test. ^#^*P* < 0.05, ^##^*P* < 0.01, ^###^*P* < 0.001 and ^####^*P* < 0.0001 compared to control group. *P < 0.05, **P < 0.01, ***P < 0.001 and ****P < 0.0001 indicate statistically significant differences between two experimental groups.

We assessed cytokine and chemokine secretion in *E. coli*-infected endometrial tissue. After 9 h, PGD_2_ treatment significantly increased TNF-α, IL-1β, IL-6, and IL-8 levels compared to the *E. coli* infection group, while IL-10 levels decreased. However, at 24 h post-infection, the expression levels of TNF-α, IL-1β, and IL-6 were markedly reduced, while the secretion of the chemokine IL-8 continued to increase, and the anti-inflammatory cytokine IL-10 was significantly upregulated (Figure 8, *P* < 0.05). These findings suggest that PGD_2_ exerts a time-dependent immunoregulatory effect during *E. coli* infection, promoting inflammatory responses at the early stage while potentially exerting anti-inflammatory effects at the later stage.

## 4 Discussion

This study explored the novel role and underlying molecular mechanisms of PGD_2_ in dairy cow endometritis. We applied PGD_2_ to *E. coli*-induced BMDMs and endometrial tissues from dairy cows, demonstrating its dual pro- and anti-inflammatory effects, depending on the stage of inflammation. Notably, the clinical features and prognosis of endometritis correlated with the duration of PGD_2_ action, irrespective of its concentration. Initially, we confirmed PGD_2_ expression in *E. coli*-induced BMDMs and then assessed its effects in a model of endometritis. Our results revealed that PGD_2_ amplifies the inflammatory response in the early stages of infection but mitigates it as the infection progresses. We measured the levels of various inflammatory mediators and tissue damage at the early (9 h) and late (24 h) stages of infection, finding consistency with cellular results. We hypothesized that PGD_2_ modulates the expression of inflammatory mediators and the killing capacity of BMDMs through different receptors at various infection stages, affecting endometrial tissue damage. However, further studies are required to fully elucidate these regulatory mechanisms.

Transcriptomic analysis revealed that the most significant changes in gene expression following *E. coli* infection in BMDMs were associated with the “inflammatory response,” with a notable increase in cytokines and PTGS2 expression. In this study, we observed increased PGD_2_ secretion following *E. coli* infection in BMDMs. Similarly, inflammation induced by *Staphylococcus aureus* led to elevated PGD_2_ levels in mouse peritoneal macrophages (43). In other contexts, excessive PGD_2_ promotes eosinophilia and elevates Th2 cytokine levels, exacerbating allergic lung inflammation in mice (44). These findings suggest that PGD_2_ plays a crucial role in regulating inflammation and immune responses. Our KEGG enrichment analysis of the top 20 *E. coli*-altered signaling pathways revealed strong associations with inflammation, particularly involving the TNF, NF-κB, cytokine-cytokine receptor interaction, and MAPK pathways. Bacterial infections typically activate inflammatory responses that protect the host by eliminating harmful stimuli and promoting tissue repair (45). However, the specific effects of PGD_2_ on the activation of NF-κB and MAPK signaling pathways, as well as its regulation of cytokine secretion during *E. coli*-induced endometritis in dairy cows remains unclear.

We observed that 24 h of PGD_2_ pretreatment followed by 6 h of *E. coli* infection increased pro-inflammatory cytokines (TNF-α, IL-1β, and IL-8) secretion in BMDMs. This response was accompanied by enhanced phosphorylation of ERK, p38, and p65 within 15–30 min. However, cytokine secretion and phosphorylation levels decreased at later stages of infection. These findings suggest that PGD_2_ initially promotes, but later suppresses inflammation, showing both pro- and anti-inflammatory effects, depending on infection duration. Similar dual roles have also been observed in other models in which PGD_2_ exacerbated allergic inflammation but protected against liver damage (46). Another study showed that PGD_2_ suppresses inflammation by inhibiting NF-κB kinase, leading to reduced secretion of pro-inflammatory cytokines (IL-1β, IL-6, TNF-α) and iNOS expression in macrophages (47). Further, PGD_2_-DP_1_ signaling plays a protective role in *Helicobacter pylori*-induced gastritis (33) and enhances antiviral immunity against respiratory syncytial virus infection (48). We hypothesized that these opposing effects are mediated by the differential activation of DP_1_ and DP_2_ receptors, which have distinct influences on cyclic AMP production, inositol phosphate conversion, and intracellular Ca^2+^ mobilization (49). Additionally, PGD_2_ upregulated both IL-6 and IL-10, regardless of the infection stage. IL-6 is a pleiotropic cytokine involved in both pro- and anti-inflammatory processes (50), whereas IL-10 inhibits pro-inflammatory cytokine production and promotes tissue repair (51, 52). Our results suggest that increased IL-10 levels in early *E. coli* infection may act as a compensatory response to innate immune overactivation, whereas its upregulation in later stages reflects PGD′_2_s anti-inflammatory action.

Macrophages are crucial for host defenses, playing key roles in phagocytosis and bacterial clearance (53). Our findings showed that PGD_2_ initially reduced the killing capacity of BMDMs during early *E. coli* infection but enhanced it in later stages. This suggests that PGD_2_ modulates the immune function by regulating the killing capacity of BMDMs, potentially mitigating *E. coli*-induced endometrial tissue injury in dairy cows. Previous evidence has linked heightened inflammatory responses and bacterial burden to lung injury (54), supporting our findings on ${\text{PG}}_{2}^{\prime }$s role in immune regulation.

HMGB-1 and HABP-2 are critical tissue damage biomarkers associated with inflammatory responses (45). HMGB-1, released by damaged cells, acts as a “necrosis marker,” enabling the immune system to identify tissue damage, initiate repair responses, and facilitate lymphocyte maturation (55). When secreted, HMGB-1 binds to various immune receptors and induces inflammation by activating NF-κB signaling, leading to the release of cytokines and recruitment of leukocytes (56). During inflammation, damaged cells release numerous endogenous molecules called DAMPs, such as heat shock proteins and low-molecular-weight hyaluronic acid (57). Notably, hyaluronic acid levels consistently increase with the severity of liver injury (58). HABP-2, a hyaluronic acid-binding protein associated with the endometrial tissue in cows, plays a vital role in endometrial function (5). The upregulation of *HMGB-1* and *HABP-2* by exogenous PGD_2_ in *E. coli*-induced BMDMs suggests that PGD_2_ enhances cellular damage and inflammatory responses. We investigated the biological impact of exogenous PGD_2_ on the intrauterine pathophysiology of dairy cows using a model of *E. coli*-induced endometritis. Previous findings indicate that high and low concentrations of PGD_2_ mediate opposing effects on cell growth, with high concentrations promoting growth and low concentrations inhibiting it (59). Therefore, we selected high (drug-level) and low (physiological-level) PGD_2_ concentrations to treat endometrial tissues from *E. coli*-infected cows and assessed their roles in dairy cow endometritis. Our results show that at 9 h post-*E. coli* infection, both concentrations of PGD_2_ significantly increased the expression of HMGB-1 and HABP-2, indicating that PGD_2_ exacerbates tissue damage and sustains inflammation. However, after 24 h, PGD_2_ significantly downregulated the expression of DAMPs in the infected bovine endometrial tissues, suggesting a reduction in inflammation and alleviation of tissue damage. Morphological observations yielded consistent results, showing that PGD_2_ exacerbated tissue damage at 9 h and alleviated it at 24 h post-*E. coli* infection. These results suggest that PGD_2_ may exhibit a pro-inflammatory effect during the initial phases of endometrial infection in *E. coli*-infected cows, followed by an anti-inflammatory effect in the later stages of infection, regardless of the PGD_2_ concentration. This phenomenon may result from PGD_2_ activating different receptors at different stages of endometritis in dairy cows. Supporting this hypothesis, maternal inflammation has been shown to exacerbate inflammatory responses, oxidative stress, and neuronal apoptosis through the activation of the COX-2-PGD_2_-DP_2_ pathway, thereby increasing the susceptibility of the offspring to brain injury (60). Systemic knockout of the DP_2_ receptor in mice moderately attenuates inflammation-induced kidney injury (61). Similarly, ${\text{DP}}_{2}^{-/-}$ mice modestly attenuate inflammation-induced kidney injury, demonstrating D_2_′s broader role in mediating pro-inflammatory damage. In contrast to the detrimental effects mediated by DP_2_, the PGD_2_-DP_1_ signaling pathway offers protective effects in different contexts. For instance, DP_1_ signaling protects against aluminum overload-induced neuronal damage in primary cultured rat hippocampal cells (62), and PGD_2_ shields neurons from glutamate toxicity or ischemia-reperfusion injury via DP_1_ receptor activation (63). The PGD_2_-DP_1_ pathway is also protective in conditions such as acute lung injury and bovine endometritis (37, 64). Therefore, PGD_2_ cannot be strictly classified as pro-inflammatory or anti-inflammatory, and its effects depend on the disease stage, cell type, and specific synthase and receptor interactions.

Previous research has demonstrated that inflammatory cytokines TNF-α or IL-1 can stimulate monocyte-macrophages to release HMGB-1, which, in turn, stimulates the secretion of inflammatory cytokines and induces the chemotaxis of neutrophils (65). In this study, assessment of cytokine secretion revealed that exogenous PGD_2_ increased the production of pro-inflammatory cytokines and chemokines while diminishing the secretion of anti-inflammatory cytokines in tissues during the early stages of infection. Conversely, diametrically opposite outcomes were observed during the later stages of infection. This highlights the multifaceted roles of PGD_2_ throughout the course of infection. The activation of this cytokine and chemokine cascade ultimately triggers an inflammatory response, leading to organ damage if left unchecked (54). Based on these findings, we hypothesized that PGD_2_ initially binds to the DP_2_ receptor in *E. coli*-infected tissues and cells, thereby increasing inflammatory responses and exacerbating tissue damage. Subsequently, PGD_2_ may bind to the DP_1_ receptor, exerting an anti-inflammatory effect that mitigates tissue damage by downregulating pro-inflammatory mediators and upregulating IL-10 secretion during *E. coli* infection.

By thoroughly exploring the dual role of PGD_2_ and its mechanism in endometritis in dairy cows, we can provide a theoretical basis for the development of safer and more effective treatments. PGD_2_ is expected to serve as a new therapeutic target to improve uterine health in dairy cows, thereby safeguarding the quality and safety of dairy products.

## 5 Conclusions

**Figure 9:**
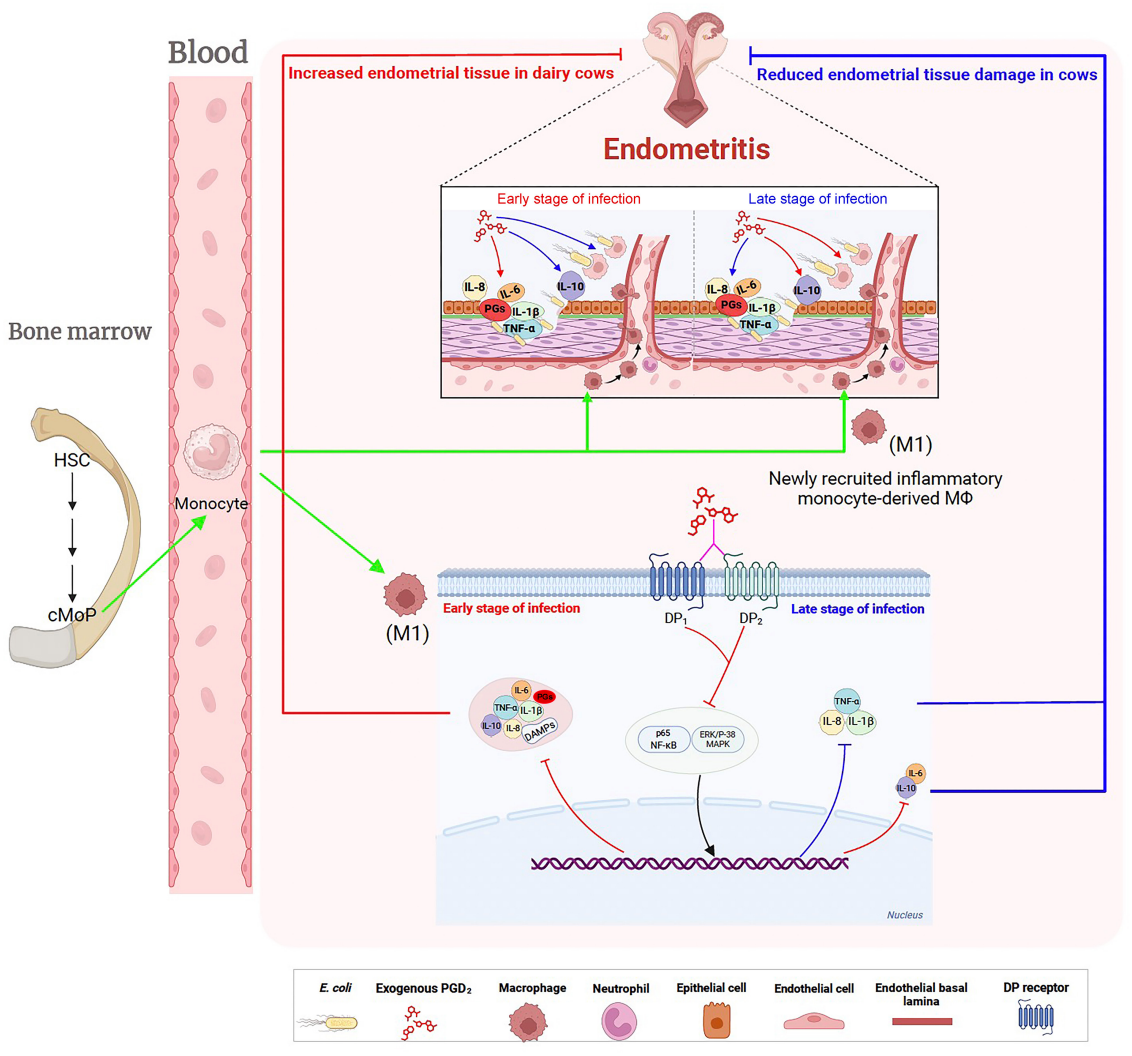
Regulatory effects of exogenous PGD_2_ on *Escherichia coli*-induced endometritis in dairy cows. Postpartum, *E. coli* invades the uterus, triggering the production of inflammatory mediators, including prostaglandins. This response recruits and activates hematopoietic cells, particularly macrophages, which differentiate into M1 macrophages to eliminate the invading *E. coli*. PGD_2_ binds to two distinct receptors, exacerbating endometrial tissue damage in the early stages of *E. coli*-induced endometritis in dairy cows by enhancing signaling pathways, increasing pro-inflammatory cytokine production, and reducing the killing capacity of BMDMs. However, in the later stages of infection, PGD_2_ exhibits a protective role. Green arrows indicate activation, migration, the red lines indicate up-regulation, the blue lines indicate down-regulation, pink lines (?) indicate recognition or interaction. HSC, hematopoietic stem cell; cMoP, common monocyte progenitor; DAMPs, damage-associated molecular patterns.

This study highlights the key role of PGD_2_ in the response to *E. coli*-induced bovine endometritis. Our findings demonstrate the different roles of exogenous PGD_2_ throughout the infection process and its molecular mechanisms (Figure 9): in the early stages, PGD_2_ enhances the inflammatory response, while later, it increases the bactericidal capacity of BMDMs against *E. coli* by reducing pro-inflammatory cytokines and chemokines, promoting anti-inflammatory factor production, and decreasing DAMPs expression, ultimately alleviating endometrial damage. Importantly, these results not only advance our understanding of the immunomodulatory role of PGD_2_ in the context of uterine inflammation but also have broad implications for improving the reproductive health of dairy cows. By elucidating a potential non-antibiotic pathway for controlling inflammation and enhancing host defense, this study provides a promising foundation for the development of novel therapeutic strategies aimed at reducing antibiotic dependence. Such strategies are critical for promoting animal welfare, improving herd fertility, and ensuring the long-term sustainability and productivity of the dairy industry.

## Data Availability

The datasets generated and/or analyzed during the current study are publicly available in the Gene Expression Omnibus (GEO) repository at NCBI under accession number GSE275904, which can be accessed at the following link: https://www.ncbi.nlm.nih.gov.
