# Supplementary material for: Exogenous prostaglandin D2 as a modulator in bovine endometritis: implications for reducing antibiotic use in dairy cattle
Source: Front Vet Sci. 2025 Aug 12;12:1618203. doi: 10.3389/fvets.2025.1618203 (PMC12379058; doi:10.3389/fvets.2025.1618203)
Supplement: Supplementary file 1 [file Image_1.pdf]

Regulation of exogenous prostaglandin D_2 in *Escherichia coli*-induced bovine bone marrow-derived macrophages and endometrial tissue

Supplemental Figure 1. Identification of M1 bone marrow-derived macrophages (BMDMs).

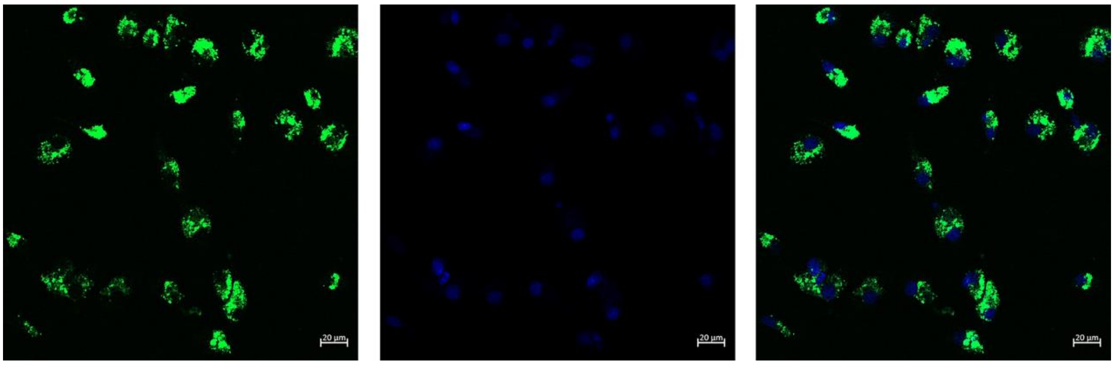

CD80 is a surface marker expressed on macrophages. The BMDMs were subjected to immunofluorescence staining for CD80 (green) and nuclear (DAPI, blue). Merge represents the composite picture of target protein and nuclear. The immunofluorescence staining was imaged by fluorescence microscopy (Zeiss LSM 800 laser, ×100 magnification).

Supplemental Figure 2. Selection of PGD_2 concentrations in *E. coli*-induced BMDMs.

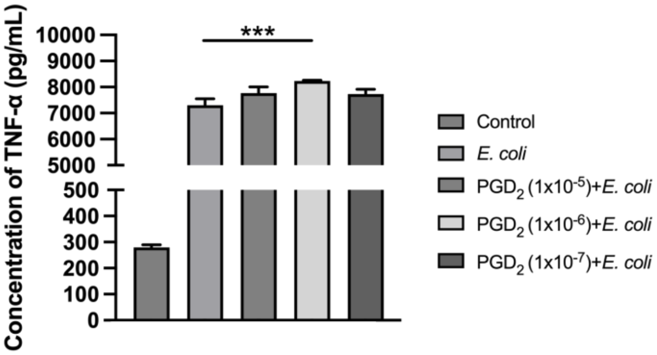

The effects of PGD_2 on TNF-α secretion in *E. coli*-induced BMDMs. Results were expressed as the mean ± SD and were analyzed by one-way ANOVA followed by Tukey's multiple comparisons test (n = 3). $^{*}P < 0.05$, $^{**}P < 0.01$, $^{***}P < 0.001$ and $^{****}P < 0.0001$.

Supplemental Figure 3. Selection of PGD_2 concentrations in *E. coli*-induced endometrial tissues.

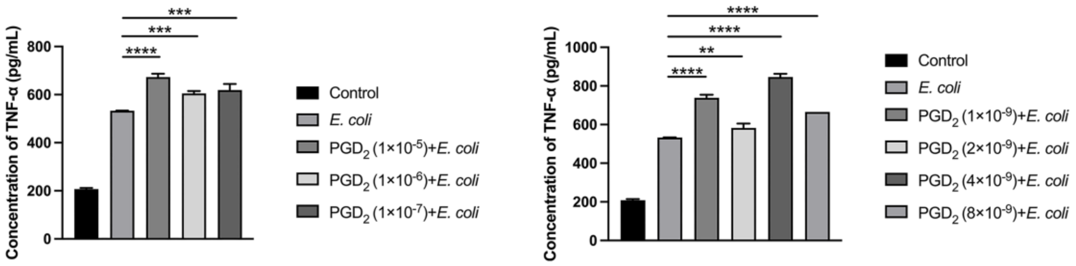

PGD_2 on TNF-α secretion in *E. coli*-induced endometrial tissues. Results were expressed as the mean ± SD and were analyzed by one-way ANOVA followed by Tukey's multiple comparisons test (n = 3). *$P < 0.05$, **$P < 0.01$, ***$P < 0.001$ and ****$P < 0.0001$.
